# Supplementary material for: Emergence of a Reassortant 2.3.4.4b Highly Pathogenic H5N1 Avian Influenza Virus Containing H9N2 PA Gene in Burkina Faso, West Africa, in 2021
Source: Viruses. 2022 Aug 27;14(9):1901. doi: 10.3390/v14091901 (PMC9504354; doi:10.3390/v14091901)
Supplement: Supplementary file 1 [file viruses-14-01901-s001.zip › Table S1.pdf]

Table S1. GISAID's EpiFlu™ Database acknowledgment table.

We gratefully acknowledge the authors, originating and submitting laboratories of the sequences from GISAID's EpiFlu™ Database on which this research is based in part. The list is detailed below.								
All submitters of data may be contacted directly via www.gisaid.org								
**Segment ID**	**Segment**	**Country**	**Collection date**	**Isolate-ID**	**Isolate_name**	**Originating Lab**	**Submitting Lab**	**Authors**
EPI1942914	HA	Belgium	2020-Dec-26	EPI_ISL_7622862	A/Anser_brachyrhynchus/Belgium/151/2020	Sciensano - Animal Infectious Diseases	Sciensano, Department of Animal Infectious Diseases	Van Borm, Steven; Vandenbussche, Frank; Roupie, Virginie; Lambrecht, Benedicte; Steensels, Mieke
EPI1848934	HA	United Kingdom	2021-Feb-10	EPI_ISL_1123361	A/pheasant/Scotland/000348/2021	Animal and Plant Health Agency (APHA)	Animal and Plant Health Agency (APHA)	
EPI1849453	HA	Netherlands	2020-Nov-10	EPI_ISL_1139102	A/swan/Netherlands/20017772-002/2020	Wageningen Bioveterinary Research	Wageningen Bioveterinary Research	Beerens, Nancy; Harders, Frank; Pritz-Verschuren, Sylvia; Roose, Marit; Germeraad, Evelien; Engelsma, Marc; Bossers, Alex; Heutink, Rene
EPI1924123	HA	United Kingdom	2021-Oct-24	EPI_ISL_5804708	A/mute_swan/England/053054/2021	Animal and Plant Health Agency (APHA)	Animal and Plant Health Agency (APHA)	
EPI1938828	HA	Sweden	2021-Nov-08	EPI_ISL_7050532	A/western_jackdaw/Sweden/SVA211111SZ0376/FB004483/2021	National Veterinary Institute, SVA	National Veterinary Institute	
EPI1807259	HA	Netherlands	2020-Oct-16	EPI_ISL_603135	A/Eurasian_Wigeon/Netherlands/5/2020	Erasmus Medical Center	Erasmus Medical Center	
EPI1933031	HA	Egypt	2020-Dec-23	EPI_ISL_6816054	A/chicken/Egypt/CAI11/2020		Import from public-domain	Khalil,H.S.; Abd El-Hamid,H.S.; Ellakany,H.F.; Elbestawy,A.R.; Mohamed,A.S.A.
EPI1933029	HA	Egypt	2020-Oct-08	EPI_ISL_6816052	A/chicken/Egypt/MEN6/2020		Import from public-domain	Khalil,H.S.; Abd El-Hamid,H.S.; Ellakany,H.F.; Elbestawy,A.R.; Mohamed,A.S.A.
EPI1933028	HA	Egypt	2020-Feb-14	EPI_ISL_6816051	A/chicken/Egypt/GIZ4/2020		Import from public-domain	Khalil,H.S.; Abd El-Hamid,H.S.; Ellakany,H.F.; Elbestawy,A.R.; Mohamed,A.S.A.
EPI1904329	HA	Egypt	2020-Sep-01	EPI_ISL_4072778	A/chicken/Egypt/v1526/2020		Import from public-domain	El-Hoseny,M.M.; Hagag,N.M.; Adel,A.; EL-Zanaty,A.; Seleem,K.; Arafa,A.; Shahien,M.
EPI1848838	HA	United Kingdom	2020-Dec-16	EPI_ISL_1123263	A/chicken/Scotland/043405/2020	Animal and Plant Health Agency (APHA)	Animal and Plant Health Agency (APHA)	
EPI1846305	HA	United Kingdom	2021-Jan-05	EPI_ISL_996003	A/chicken/Northern_Ireland/2021-000067_21VIR114-19/2021	AFBI - Agri-Food & Bioscience Institute	Istituto Zooprofilattico Sperimentale Delle Venezie	McMenamy, MJ.; Harkin, V.; Lemon, K.; Zecchin, B.; Fusaro, A.; Schivo, A.; Salviato, A.; Pastori, A.; Monne, I.; Terregino, C.

EPI1933027	HA	Egypt	2020-Jan-15	EPI_ISL_6816050	A/Duck/Egypt/BEH2/2020		Import from public-domain	Khalil,H.S.; Abd El-Hamid,H.S.; Ellakany,H.F.; Elbestawy,A.R.; Mohamed,A.S.A.
EPI1850269	HA	Netherlands	2021-Feb-01	EPI_ISL_1224989	A/common_buzzard/Netherlands/21022834-002/2021	Wageningen Bioveterinary Research	Wageningen Bioveterinary Research	Beerens, Nancy; Harders, Frank; Pritz-Verschuren, Sylvia; Roose, Marit; Germeraad, Evelien; Engelsma, Marc; Bossers, Alex; Heutink, Rene
EPI1945522	HA	Finland	2021-May-01	EPI_ISL_7778773	A/barnacle_goose/Finland/6247_21VIR7689-6/2021	Finnish Food Authority	Istituto Zooprofilattico Sperimentale Delle Venezie	Tammiranta, N.; Kantala, T.; Laamanen, I.; Gadd, T.; Zecchin, B.; Fusaro, A.; Schivo, A.; Salviato, A.; Palumbo, E.; Milani, A.; Giussani, E.; Pastori, A.; Monne, I.; Terregino, C.
EPI1945485	HA	Finland	2021-Aug-01	EPI_ISL_7778768	A/Eurasian_eagle-owl/Finland/10617_21VIR7689-15/2021	Finnish Food Authority	Istituto Zooprofilattico Sperimentale Delle Venezie	Tammiranta, N.; Kantala, T.; Laamanen, I.; Gadd, T.; Zecchin, B.; Fusaro, A.; Schivo, A.; Salviato, A.; Palumbo, E.; Milani, A.; Giussani, E.; Pastori, A.; Monne, I.; Terregino, C.
EPI1945469	HA	Finland	2021-Aug-01	EPI_ISL_7778766	A/European_herring_gull/Finland/9722_21VIR7689-13/2021	Finnish Food Authority	Istituto Zooprofilattico Sperimentale Delle Venezie	Tammiranta, N.; Kantala, T.; Laamanen, I.; Gadd, T.; Zecchin, B.; Fusaro, A.; Schivo, A.; Salviato, A.; Palumbo, E.; Milani, A.; Giussani, E.; Pastori, A.; Monne, I.; Terregino, C.
EPI1945381	HA	Estonia	2021-May-16	EPI_ISL_7778755	A/white-tailed_eagle/Estonia/TA2111864-2_21VIR7512-6/2021	Estonian Veterinary and Food Laboratory	Istituto Zooprofilattico Sperimentale Delle Venezie	Nurmoja, I.; Vilem, A.; Juurik, T.; Zecchin, B.; Fusaro, A.; Schivo, A.; Salviato, A.; Palumbo, E.; Milani, A.; Giussani, E.; Pastori, A.; Monne, I.; Terregino, C.
EPI1883621	HA	Romania	2021-Apr-08	EPI_ISL_3102081	A/mute_swan/Romania/11981-1_21VIR3163-5/2021	Istituto Zooprofilattico Sperimentale delle Venezie, EU/OIE/Reference Laboratory and FAO Reference Centre for AI and ND	Istituto Zooprofilattico Sperimentale Delle Venezie	Onita, I.; Neicut, A.; Raluca, B.; Razvan, M.; Florica, B.; Zecchin, B.; Fusaro, A.; Giussani, E.; Schivo, A.; Salviato, A.; Monne, I.; Terregino, C.
EPI1812367	HA	Netherlands	2020-Oct-28	EPI_ISL_632314	A/greylag_goose/Netherlands/20016582-004/2020	Wageningen Bioveterinary Research	Wageningen Bioveterinary Research	Beerens, Nancy; Harders, Frank; Verschuren-Pritz, Sylvia; Roose, Marit; Germeraad, Evelien; Engelsma, Marc; Bossers, Alex; Heutink, Rene
EPI1848910	HA	United Kingdom	2020-Nov-12	EPI_ISL_1123358	A/brent_goose/England/095684/2020	Animal and Plant Health Agency (APHA)	Animal and Plant Health Agency (APHA)	

EPI1848902	HA	United Kingdom	2020-Nov-08	EPI_ISL_1123357	A/brent_goose/England/233339/2020	Animal and Plant Health Agency (APHA)	Animal and Plant Health Agency (APHA)	
EPI1850253	HA	Netherlands	2021-Jan-31	EPI_ISL_1224946	A/barnacle_goose/Netherlands/21022611-001/2021	Wageningen Bioveterinary Research	Wageningen Bioveterinary Research	Beerens, Nancy; Harders, Frank; Pritz-Verschuren, Sylvia; Roose, Marit; Germeraad, Evelien; Engelsma, Marc; Bossers, Alex; Heutink, Rene
EPI1811572	HA	Germany	2020-Oct-28	EPI_ISL_614400	A/barnacle_goose/Germany-SH/AI02167/2020	Landeslabor Schleswig-Holstein	Friedrich-Loeffler-Institut	
EPI1848662	HA	Russian Federation	2020-Oct-17	EPI_ISL_1114745	A/chicken/Kostroma/304-10/2020	State Research Center of Virology and Biotechnology (VECTOR)	State Research Center of Virology and Biotechnology (VECTOR)	Natalia,Goncharova; Ivan,Susloparov; Natalia,Kolosova; Alexey,Danilenko; Juliya,Bulanovich; Vasiliy,Marchenko; Alexander,Ryzhikov
EPI1848654	HA	Russian Federation	2020-Oct-17	EPI_ISL_1114742	A/chicken/Kostroma/304-08/2020	State Research Center of Virology and Biotechnology (VECTOR)	State Research Center of Virology and Biotechnology (VECTOR)	Natalia,Goncharova; Ivan,Susloparov; Natalia,Kolosova; Alexey,Danilenko; Juliya,Bulanovich; Vasiliy,Marchenko; Alexander,Ryzhikov
EPI1848646	HA	Russian Federation	2020-Oct-17	EPI_ISL_1114741	A/chicken/Kostroma/304-06/2020	State Research Center of Virology and Biotechnology (VECTOR)	State Research Center of Virology and Biotechnology (VECTOR)	Natalia,Goncharova; Ivan,Susloparov; Natalia,Kolosova; Alexey,Danilenko; Juliya,Bulanovich; Vasiliy,Marchenko; Alexander,Ryzhikov
EPI1848614	HA	Russian Federation	2020-Sep-26	EPI_ISL_1114736	A/chicken/Tyumen/302-02/2020	State Research Center of Virology and Biotechnology (VECTOR)	State Research Center of Virology and Biotechnology (VECTOR)	Natalia,Goncharova; Ivan,Susloparov; Natalia,Kolosova; Alexey,Danilenko; Juliya,Bulanovich; Vasiliy,Marchenko; Alexander,Ryzhikov

EPI1848606	HA	Russian Federation	2020-Sep-26	EPI_ISL_1114735	A/chicken/Tyumen/302-01/2020	State Research Center of Virology and Biotechnology (VECTOR)	State Research Center of Virology and Biotechnology (VECTOR)	Natalia,Goncharova; Ivan,Susloparov; Natalia,Kolosova; Alexey,Danilenko; Juliya,Bulanovich; Vasiliy,Marchenko; Alexander,Ryzhikov
EPI1813145	HA	Russian Federation	2020-Aug-17	EPI_ISL_644125	A/goose/Omsk/0111/2020	State Research Center of Virology and Biotechnology (VECTOR)	State Research Center of Virology and Biotechnology (VECTOR)	Natalia,Goncharova; Ivan,Susloparov; Natalia,Kolosova; Alexey,Danilenko; Juliya,Bulanovich; Vasiliy,Marchenko; Alexander,Ryzhikov
EPI1813297	HA	Russian Federation	2020-Aug-17	EPI_ISL_644144	A/duck/Omsk/0077/2020	State Research Center of Virology and Biotechnology (VECTOR)	State Research Center of Virology and Biotechnology (VECTOR)	Natalia,Goncharova; Ivan,Susloparov; Natalia,Kolosova; Alexey,Danilenko; Juliya,Bulanovich; Vasiliy,Marchenko; Alexander,Ryzhikov
EPI1813281	HA	Russian Federation	2020-Aug-17	EPI_ISL_644142	A/duck/Omsk/0075/2020	State Research Center of Virology and Biotechnology (VECTOR)	State Research Center of Virology and Biotechnology (VECTOR)	Natalia,Goncharova; Ivan,Susloparov; Natalia,Kolosova; Alexey,Danilenko; Juliya,Bulanovich; Vasiliy,Marchenko; Alexander,Ryzhikov
EPI1812375	HA	Netherlands	2020-Nov-02	EPI_ISL_632315	A/eurasian_teal/Netherlands/20016896-013/2020	Wageningen Bioveterinary Research	Wageningen Bioveterinary Research	Beerens, Nancy; Harders, Frank; Verschuren-Pritz, Sylvia; Roose, Marit; Germeraad, Evelien; Engelsma, Marc; Bossers, Alex; Heutink, Rene
EPI1813129	HA	Russian Federation	2020-Aug-13	EPI_ISL_644123	A/turkey/Omsk/0003/2020	State Research Center of Virology and Biotechnology (VECTOR)	State Research Center of Virology and Biotechnology (VECTOR)	Natalia,Goncharova; Ivan,Susloparov; Natalia,Kolosova; Alexey,Danilenko; Juliya,Bulanovich; Vasiliy,Marchenko; Alexander,Ryzhikov
EPI1843642	HA	Italy	2020-Nov-23	EPI_ISL_956412	A/greater_white-fronted_goose/Italy/20VIR8073-4/2020	Istituto Zooprofilattico Sperimentale delle Venezie, EU/OIE/Reference Laboratory and FAO	Istituto Zooprofilattico Sperimentale Delle Venezie	Zecchin, B.; Fusaro, A.; Milani, A.; Schivo, A.; Salviato, A.; Pastori, A.; Zamperin, G.; Monne, I.; Terregino, C.

						Reference Centre for AI and ND		
EPI1858284	HA	Slovakia	2021-Jan-29	EPI_ISL_1665261	A/mute_swan/Slovakia/Pah15_21VIR1086-3/2021	Istituto Zooprofilattico Sperimentale delle Venezie, EU/OIE/Reference Laboratory and FAO Reference Centre for AI and ND	Istituto Zooprofilattico Sperimentale Delle Venezie	Dirb?kov?, Z.; Tin?k, M.; Zecchin, B.; Fusaro, A.; Pastori, A.; Schivo, A.; Salviato, A.; Monne, I.; Terregino, C.
EPI1858260	HA	Austria	2021-Feb-10	EPI_ISL_1665258	A/mute_swan/Austria/21013162_21VIR1085-5/2021	Istituto Zooprofilattico Sperimentale delle Venezie, EU/OIE/Reference Laboratory and FAO Reference Centre for AI and ND	Istituto Zooprofilattico Sperimentale Delle Venezie	Wodak, E.; Revilla Fern?ndez, S.; Schmoll, F.; Zecchin, B.; Fusaro, A.; Pastori, A.; Schivo, A.; Salviato, A.; Monne, I.; Terregino, C.
EPI1930936	HA	Egypt	2021-Nov-01	EPI_ISL_6781491	A/Turkey/Egypt/A2/2021		Import from public-domain	Hagag,N.; ElHusseiny,M.; Selim,A.; Zanaty,A.; Arafa,A.
EPI1837949	HA	United Kingdom	2020-Dec-02	EPI_ISL_710505	A/turkey/England/038115/2020	Animal and Plant Health Agency (APHA)	Animal and Plant Health Agency (APHA)	
EPI1995204	HA	Niger	2022-Jan-01	EPI_ISL_11007545	A/quail/Niger/22VIR1409-30/2022	Beijing Genomics Institute (BGI)	Istituto Zooprofilattico Sperimentale delle Venezie	Souley, M.M.; Yaou, B.; Amadou, H.; Haido, A.M.; Issiako, A.; Alassane, A.; Barbierato, G.; Zecchin, B.; Fusaro, A.; Schivo, A.; Salviato, A.; Palumbo, E.; Giussani, E.; Monne, I.; Terregino, C.
EPI1995196	HA	Niger	2022-Jan-01	EPI_ISL_11007544	A/quail/Niger/22VIR1409-28/2022	Beijing Genomics Institute (BGI)	Istituto Zooprofilattico Sperimentale delle Venezie	Souley, M.M.; Yaou, B.; Amadou, H.; Haido, A.M.; Issiako, A.; Alassane, A.; Barbierato, G.; Zecchin, B.; Fusaro, A.; Schivo, A.; Salviato, A.; Palumbo, E.; Giussani, E.; Monne, I.; Terregino, C.
EPI1859803	HA	Netherlands	2021-Mar-17	EPI_ISL_2174084	A/common_murre/Netherlands/21025491-002/2021	Wageningen Bioveterinary Research	Wageningen Bioveterinary Research	Beerens, Nancy; Harders, Frank; Pritz-Verschuren, Sylvia; Roose, Marit; Germeraad, Evelien; Engelsma, Marc; Bossers, Alex; Heutink, Rene
EPI1877873	HA	Poland	2021-Apr-20	EPI_ISL_2681045	A/white_stork/Poland/MB391/2021	National Veterinary Research Institut Poland, PIWet-PIB	National Veterinary Research Institut Poland, PIWet-PIB	Edyta, Swieton; Kamila, Dziadek; Krzysztof, Smietanka

EPI1995220	HA	Niger	2022-Jan-01	EPI_ISL_11007547	A/chicken/Niger/22VIR1409-9/2022	Beijing Genomics Institute (BGI)	Istituto Zooprofilattico Sperimentale delle Venezie	Souley, M.M.; Yaou, B.; Amadou, H.; Haido, A.M.; Issiako, A.; Alassane, A.; Barbierato, G.; Zecchin, B.; Fusaro, A.; Schivo, A.; Salviato, A.; Palumbo, E.; Giussani, E.; Monne, I.; Terregino, C.
EPI1995212	HA	Niger	2022-Jan-01	EPI_ISL_11007546	A/chicken/Niger/22VIR1409-5/2022	Beijing Genomics Institute (BGI)	Istituto Zooprofilattico Sperimentale delle Venezie	Souley, M.M.; Yaou, B.; Amadou, H.; Haido, A.M.; Issiako, A.; Alassane, A.; Barbierato, G.; Zecchin, B.; Fusaro, A.; Schivo, A.; Salviato, A.; Palumbo, E.; Giussani, E.; Monne, I.; Terregino, C.
EPI1995188	HA	Niger	2022-Jan-01	EPI_ISL_11007543	A/chicken/Niger/22VIR1409-23/2022	Beijing Genomics Institute (BGI)	Istituto Zooprofilattico Sperimentale delle Venezie	Souley, M.M.; Yaou, B.; Amadou, H.; Haido, A.M.; Issiako, A.; Alassane, A.; Barbierato, G.; Zecchin, B.; Fusaro, A.; Schivo, A.; Salviato, A.; Palumbo, E.; Giussani, E.; Monne, I.; Terregino, C.
EPI1995180	HA	Niger	2022-Jan-01	EPI_ISL_11007542	A/chicken/Niger/22VIR1409-13/2022	Beijing Genomics Institute (BGI)	Istituto Zooprofilattico Sperimentale delle Venezie	Souley, M.M.; Yaou, B.; Amadou, H.; Haido, A.M.; Issiako, A.; Alassane, A.; Barbierato, G.; Zecchin, B.; Fusaro, A.; Schivo, A.; Salviato, A.; Palumbo, E.; Giussani, E.; Monne, I.; Terregino, C.
EPI1896085	HA	Nigeria	2021-Feb-12	EPI_ISL_4061491	A/chicken/Nigeria/VRD21-98_21VIR2288-6/2021		Import from public-domain	Shittu,I.; Meseko,C.; Nwosuh,C.; Muhammad,M.; Alabi,O.; Tassoni,L.; Schivo,A.; Salviato,A.; Edoardo,G.; Zecchin,B.; Fusaro,A.; Monne,I.
EPI1898064	HA	Nigeria	2021-Feb-25	EPI_ISL_4061490	A/chicken/Nigeria/VRD21-88_21VIR2288-8/2021		Import from public-domain	Shittu,I.; Meseko,C.; Nwosuh,C.; Muhammad,M.; Alabi,O.; Tassoni,L.; Schivo,A.; Salviato,A.; Edoardo,G.; Zecchin,B.; Fusaro,A.; Monne,I.
EPI1896077	HA	Nigeria	2021-Feb-11	EPI_ISL_4061488	A/chicken/Nigeria/VRD21-53B_21VIR2288-5/2021		Import from public-domain	Shittu,I.; Meseko,C.; Nwosuh,C.; Muhammad,M.; Alabi,O.; Tassoni,L.; Schivo,A.; Salviato,A.; Edoardo,G.; Zecchin,B.; Fusaro,A.; Monne,I.

EPI1896075	HA	Nigeria	2021-Feb-05	EPI_ISL_4061486	A/chicken/Nigeria/VRD21-37_21VIR2288-2/2021		Import from public-domain	Shittu,I.; Meseko,C.; Nwosuh,C.; Muhammad,M.; Alabi,O.; Tassoni,L.; Schivo,A.; Salviato,A.; Edoardo,G.; Zecchin,B.; Fusaro,A.; Monne,I.
EPI1896066	HA	Nigeria	2021-Mar-01	EPI_ISL_4061485	A/chicken/Nigeria/VRD21-109_21VIR2370-425/2021		Import from public-domain	Shittu,I.; Meseko,C.; Nwosuh,C.; Muhammad,M.; Alabi,O.; Tassoni,L.; Schivo,A.; Salviato,A.; Edoardo,G.; Zecchin,B.; Fusaro,A.; Monne,I.
EPI1896058	HA	Nigeria	2021-Mar-01	EPI_ISL_4061484	A/chicken/Nigeria/VRD21-102_21VIR2370-424/2021		Import from public-domain	Shittu,I.; Meseko,C.; Nwosuh,C.; Muhammad,M.; Alabi,O.; Tassoni,L.; Schivo,A.; Salviato,A.; Edoardo,G.; Zecchin,B.; Fusaro,A.; Monne,I.
EPI1896050	HA	Nigeria	2021-Mar-01	EPI_ISL_4061483	A/chicken/Nigeria/VRD21-100_21VIR2370-423/2021		Import from public-domain	Shittu,I.; Meseko,C.; Nwosuh,C.; Muhammad,M.; Alabi,O.; Tassoni,L.; Schivo,A.; Salviato,A.; Edoardo,G.; Zecchin,B.; Fusaro,A.; Monne,I.
EPI1896042	HA	Nigeria	2021-Feb-05	EPI_ISL_4061482	A/chicken/Nigeria/VRD21-035B_21VIR2288-1/2021		Import from public-domain	Shittu,I.; Meseko,C.; Nwosuh,C.; Muhammad,M.; Alabi,O.; Tassoni,L.; Schivo,A.; Salviato,A.; Edoardo,G.; Zecchin,B.; Fusaro,A.; Monne,I.
EPI1896034	HA	Nigeria	2021-Feb-10	EPI_ISL_4061481	A/chicken/Nigeria/VRD21-43_21VIR2288-4/2021		Import from public-domain	Shittu,I.; Meseko,C.; Nwosuh,C.; Muhammad,M.; Alabi,O.; Tassoni,L.; Schivo,A.; Salviato,A.; Edoardo,G.; Zecchin,B.; Fusaro,A.; Monne,I.
EPI1866465	HA	Senegal	2020-Dec-23	EPI_ISL_2276070	A/chicken/Senegal/21VIR1084-5/2021	Istituto Zooprofilattico Sperimentale delle Venezie, EU/OIE/Reference Laboratory and FAO Reference Centre for AI and ND	Istituto Zooprofilattico Sperimentale Delle Venezie	Lo, F.T.; Diallo, A.A.; Ba, R.O.; Diouf, M.; Diop, A.; Samb, Y.N.; Diouf, M.; Diop, M.; Lo, M.M.; Diouf, M.N.; Zecchin, B.; Tassoni, L.; Fusaro, A.; Pastori, A.; Monne, I.; Terregino, C.
EPI1866457	HA	Senegal	2020-Dec-23	EPI_ISL_2276069	A/chicken/Senegal/21VIR1084-4/2021	Istituto Zooprofilattico Sperimentale delle Venezie, EU/OIE/Reference Laboratory and FAO Reference Centre for AI and ND	Istituto Zooprofilattico Sperimentale Delle Venezie	Lo, F.T.; Diallo, A.A.; Ba, R.O.; Diouf, M.; Diop, A.; Samb, Y.N.; Diouf, M.; Diop, M.; Lo, M.M.; Diouf, M.N.; Zecchin, B.; Tassoni, L.; Fusaro, A.; Pastori, A.; Monne, I.; Terregino, C.

EPI1866449	HA	Senegal	2020-Dec-23	EPI_ISL_2276068	A/chicken/Senegal/21VIR1084-3/2021	Istituto Zooprofilattico Sperimentale delle Venezie, EU/OIE/Reference Laboratory and FAO Reference Centre for AI and ND	Istituto Zooprofilattico Sperimentale Delle Venezie	Lo, F.T.; Diallo, A.A.; Ba, R.O.; Diouf, M.; Diop, A.; Samb, Y.N.; Diouf, M.; Diop, M.; Lo, M.M.; Diouf, M.N.; Zecchin, B.; Tassoni, L.; Fusaro, A.; Pastori, A.; Monne, I.; Terregino, C.
EPI1858204	HA	Slovakia	2021-Jan-22	EPI_ISL_1665250	A/chicken/Slovakia/Pah10_21VIR1086-5/2021	Istituto Zooprofilattico Sperimentale delle Venezie, EU/OIE/Reference Laboratory and FAO Reference Centre for AI and ND	Istituto Zooprofilattico Sperimentale Delle Venezie	Dirb?kov?, Z.; Tin?k, M.; Zecchin, B.; Fusaro, A.; Pastori, A.; Schivo, A.; Salviato, A.; Monne, I.; Terregino, C.
EPI1858196	HA	Romania	2021-Feb-23	EPI_ISL_1665249	A/chicken/Romania/10101_21VIR2044-1/2021	Istituto Zooprofilattico Sperimentale delle Venezie, EU/OIE/Reference Laboratory and FAO Reference Centre for AI and ND	Istituto Zooprofilattico Sperimentale Delle Venezie	Onita, I.; Neicut, A.; Raluca, B.; Razvan, M.; Florica, B.; Zecchin, B.; Fusaro, A.; Pastori, A.; Schivo, A.; Salviato, A.; Monne, I.; Terregino, C.
EPI1838673	HA	Netherlands	2020-Dec-14	EPI_ISL_711055	A/chicken/Netherlands/20019879-001005/2020	Wageningen Bioveterinary Research	Wageningen Bioveterinary Research	Beerens, Nancy; Harders, Frank; Pritz-Verschuren, Sylvia; Roose, Marit; Germeraad, Evelien; Engelsma, Marc; Bossers, Alex; Heutink, Rene
EPI1837899	HA	United Kingdom	2020-Nov-19	EPI_ISL_710512	A/whistling_duck/England/035643/2020	Animal and Plant Health Agency (APHA)	Animal and Plant Health Agency (APHA)	
EPI1866473	HA	Senegal	2021-Jan-23	EPI_ISL_2276071	A/great-white_pelican/Senegal/21-67_21VIR1084-8/2021	Istituto Zooprofilattico Sperimentale delle Venezie, EU/OIE/Reference Laboratory and FAO Reference Centre for AI and ND	Istituto Zooprofilattico Sperimentale Delle Venezie	Lo, F.T.; Diallo, A.A.; Ba, R.O.; Diouf, M.; Diop, A.; Samb, Y.N.; Diouf, M.; Diop, M.; Lo, M.M.; Diouf, M.N.; Zecchin, B.; Tassoni, L.; Fusaro, A.; Pastori, A.; Monne, I.; Terregino, C.
EPI1860087	HA	Spain	2020-Nov-20	EPI_ISL_2234814	A/peregrine_falcon/Spain/3365-1_21VIR1230-1/2020	Istituto Zooprofilattico Sperimentale delle Venezie, EU/OIE/Reference Laboratory and FAO Reference Centre for AI and ND	Istituto Zooprofilattico Sperimentale Delle Venezie	Ruano Ramos, M.J.; S?nchez S?nchez, A.; Zecchin, B.; Fusaro, A.; Schivo, A.; Salviato, A.; Giussani, E.; Monne, I.; Terregino, C.
EPI1860071	HA	Spain	2021-Jan-27	EPI_ISL_2234812	A/anser_anser/Spain/297-1_21VIR1230-5/2021	Istituto Zooprofilattico Sperimentale delle Venezie, EU/OIE/Reference Laboratory and FAO	Istituto Zooprofilattico Sperimentale Delle Venezie	Ruano Ramos, M.J.; S?nchez S?nchez, A.; Zecchin, B.; Fusaro, A.; Schivo, A.; Salviato, A.; Giussani, E.; Monne, I.; Terregino, C.

						Reference Centre for AI and ND		
EPI1942402	HA	Belgium	2020-Nov-13	EPI_ISL_7596571	A/Pica_pica/Belgium/12100_005/2020	Sciensano - Animal Infectious Diseases	Sciensano, Department of Animal Infectious Diseases	Van Borm, Steven; Vandenbussche, Frank; Roupie, Virginie; Lambrecht, Benedicte; Steensels, Mieke
EPI1922851	HA	Denmark	2021-Mar-01	EPI_ISL_5449305	A/barnacle_goose/Denmark/17572-1.01/2021-03-01	Statens Serumm Institute	Statens Serum Institute	Hjulsager, C
EPI1862722	HA	Netherlands	2021-May-13	EPI_ISL_2227277	A/barnacle_goose/Netherlands/21028534-002/2021	Wageningen Bioveterinary Research	Wageningen Bioveterinary Research	Beerens, Nancy; Harders, Frank; Pritz-Verschuren, Sylvia; Roose, Marit; Germeraad, Evelien; Engelsma, Marc; Bossers, Alex; Heutink, Rene
EPI1859739	HA	Netherlands	2021-Feb-22	EPI_ISL_2172528	A/barnacle_goose/Netherlands/21024066-001/2021	Wageningen Bioveterinary Research	Wageningen Bioveterinary Research	Beerens, Nancy; Harders, Frank; Pritz-Verschuren, Sylvia; Roose, Marit; Germeraad, Evelien; Engelsma, Marc; Bossers, Alex; Heutink, Rene
EPI1814675	HA	Belgium	2020-Nov-07	EPI_ISL_661313	A/Anser_albifrons/Belgium/11956_005/2020	Sciensano - Animal Infectious Diseases	Sciensano, Department of Animal Infectious Diseases	Van Borm,Steven; Mathijs,Elisabeth; Vandenbussche,Frank; van den Berg,Thierry; Lambrecht,Bénédicte; Steensels,Mieke
EPI1882551	HA	Kazakhstan	2020-Sep-22	EPI_ISL_2932612	A/goose/Kazakhstan/7-20-B-Talg-12/2020	National Veterinary Reference Center	National Center of Biotechnology Republic of Kazakhstan	Asylulan,Amirgazin; Alexandr,Shevtsov; Karibayev,Talgat; Tashkenbayev,Arman; Suraganova,Fariza; Kozhakhmetova,Tamila
EPI1942903	HA	Belgium	2020-Dec-03	EPI_ISL_7622619	A/Anser_Brachyrhynchus_Anser_Anser/Belgium/13846/2020	Sciensano - Animal Infectious Diseases	Sciensano, Department of Animal Infectious Diseases	Van Borm, Steven; Vandenbussche, Frank; Roupie, Virginie; Lambrecht, Benedicte; Steensels, Mieke
EPI1938836	HA	Sweden	2021-Nov-09	EPI_ISL_7053000	A/common_buzzard/Sweden/SVA211111SZ0376/FB004484/2021	National Veterinary Institute, SVA	National Veterinary Institute	
EPI1846297	HA	United Kingdom	2020-Dec-31	EPI_ISL_995172	A/chicken/Northern_Ireland/2020-17671_21VIR113-11/2020	AFBI - Agri-Food & Bioscience Institute	Istituto Zooprofilattico Sperimentale Delle Venezie	McMenamy, MJ.; Harkin, V.; Lemon, K.; Zecchin, B.; Fusaro, A.; Schivo, A.; Salviato, A.; Pastori, A.; Monne, I.; Terregino, C.
EPI1882548	HA	Kazakhstan	2020-Sep-15	EPI_ISL_2932609	A/duck/Kazakhstan/12-20-B-Talg-11/2020	National Veterinary Reference Center	National Center of Biotechnology Republic of Kazakhstan	Asylulan,Amirgazin; Alexandr,Shevtsov; Karibayev,Talgat; Tashkenbayev,Arman;

								Suraganova,Fariza; Kozhakhmetova,Tamila
EPI1847700	HA	Netherlands	2021-Jan-09	EPI_ISL_1048240	A/common_buzzard/Netherlands/21021396-002/2021	Wageningen Bioveterinary Research	Wageningen Bioveterinary Research	Beerens, Nancy; Harders, Frank; Pritz-Verschuren, Sylvia; Roose, Marit; Germeraad, Evelien; Engelsma, Marc; Bossers, Alex; Heutink, Rene
EPI1815142	HA	Italy	2020-Nov-21	EPI_ISL_683592	A/Eurasian_wigeon/Italy/20VIR7301-206/2020	Istituto Zooprofilattico Sperimentale delle Venezie, EU/OIE/Reference Laboratory and FAO Reference Centre for AI and ND	Istituto Zooprofilattico Sperimentale Delle Venezie	Zecchin, B.; Fusaro, A.; Pastori, A.; Milani, A.; Salviato, A.; Schivo, A.; Monne, I.; Terregino, C.
EPI1859811	HA	Netherlands	2021-Mar-22	EPI_ISL_2174728	A/barnacle_goose/Netherlands/21025769-002/2021	Wageningen Bioveterinary Research	Wageningen Bioveterinary Research	Beerens, Nancy; Harders, Frank; Pritz-Verschuren, Sylvia; Roose, Marit; Germeraad, Evelien; Engelsma, Marc; Bossers, Alex; Heutink, Rene
EPI1882563	HA	Kazakhstan	2020-Oct-09	EPI_ISL_2932683	A/chicken/Kazakhstan/1-20-B-Talg-67/2020	National Veterinary Reference Center	National Center of Biotechnology Republic of Kazakhstan	Asylulan,Amirgazin; Alexandr,Shevtsov; Karibayev,Talgat; Tashkenbayev,Arman; Suraganova,Fariza; Kozhakhmetova,Tamila
EPI1814337	HA	Russian Federation	2020-Sep-08	EPI_ISL_654833	A/chicken/Chelyabinsk/201/2020	State Research Center of Virology and Biotechnology (VECTOR)	State Research Center of Virology and Biotechnology (VECTOR)	Natalia,Goncharova; Ivan,Susloparov; Natalia,Kolosova; Alexey,Danilenko; Juliya,Bulanovich; Vasiliy,Marchenko; Alexander,Ryzhikov
