# Supplementary figures and images for: Emergence of a Reassortant 2.3.4.4b Highly Pathogenic H5N1 Avian Influenza Virus Containing H9N2 PA Gene in Burkina Faso, West Africa, in 2021
Source: Viruses. 2022 Aug 27;14(9):1901. doi: 10.3390/v14091901 (PMC9504354; doi:10.3390/v14091901)

**Figure S1**  
Maximum Likelihood phylogenetic tree  
of the PB2 gene, inferred using  
IQTree v1.6.6

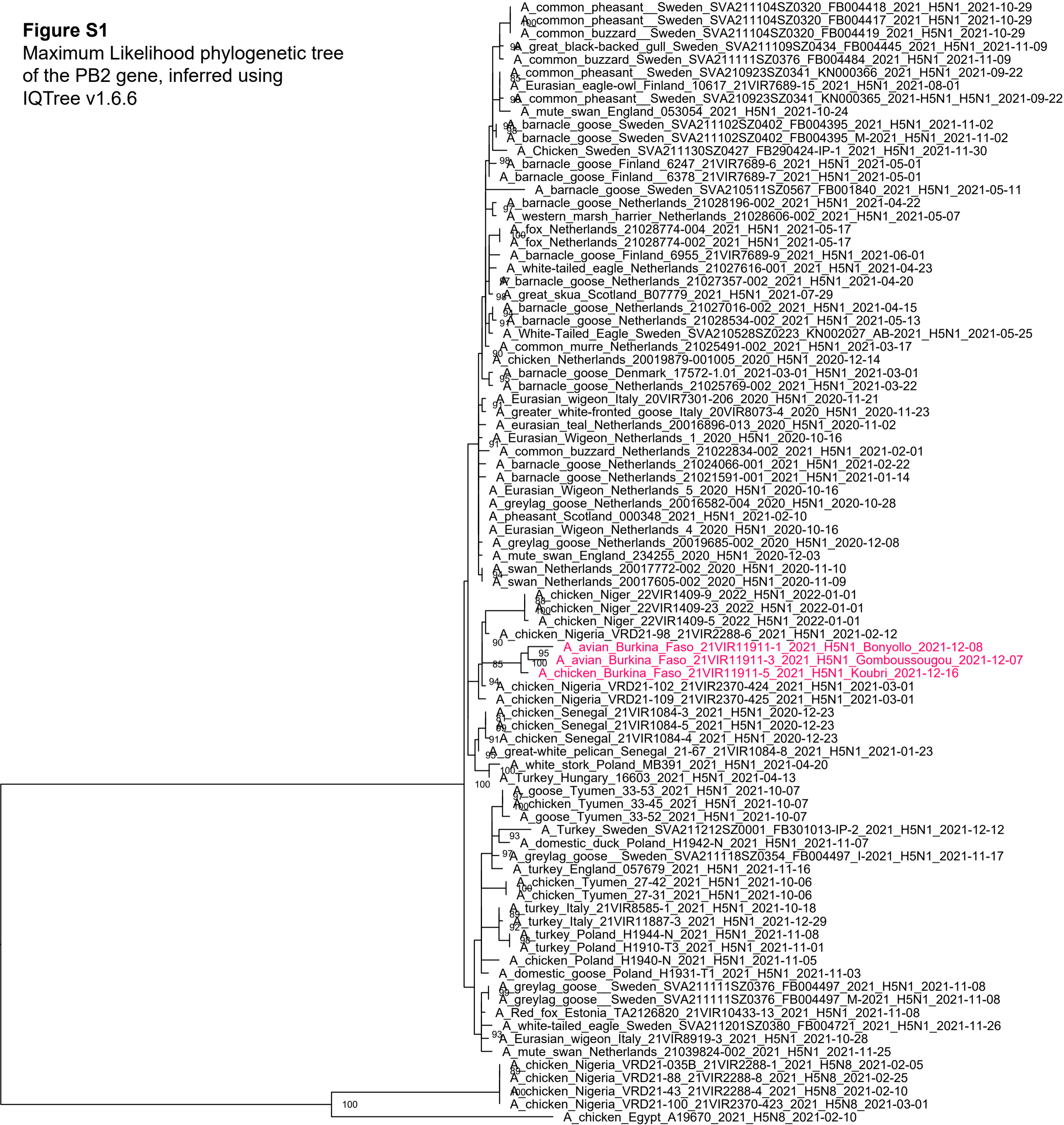

0.007

Supplement: Supplementary file 1 [file viruses-14-01901-s001.zip › Figure_S1_PB2_tree.pdf]

**Figure S2**  
Maximum Likelihood phylogenetic tree  
of the PB1 gene, inferred using  
IQTree v1.6.6

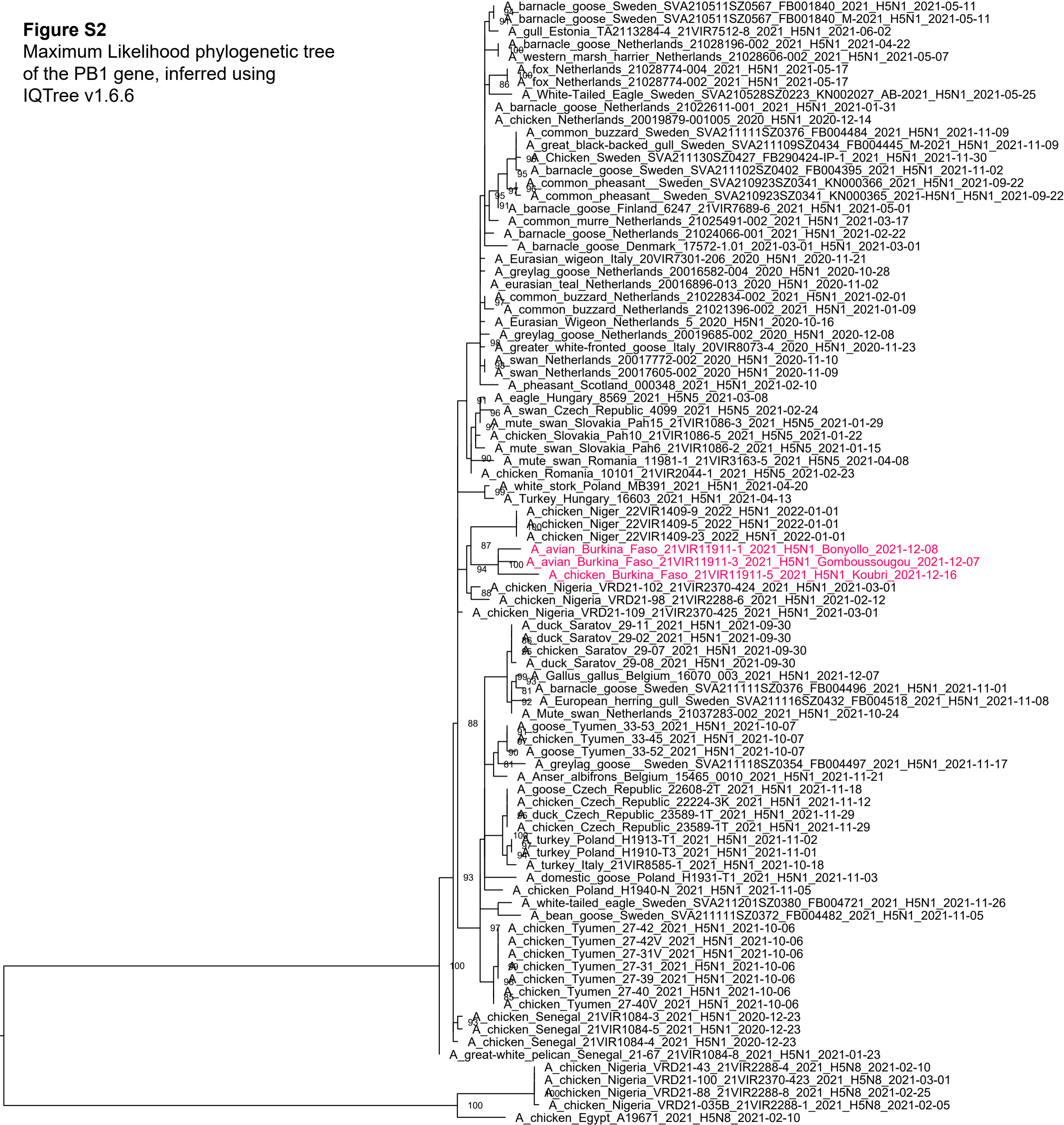

Supplement: Supplementary file 1 [file viruses-14-01901-s001.zip › Figure_S2_PB1_tree.pdf]

**Figure S3**  
Maximum Likelihood phylogenetic tree  
of the NP gene, inferred using  
IQTree v1.6.6

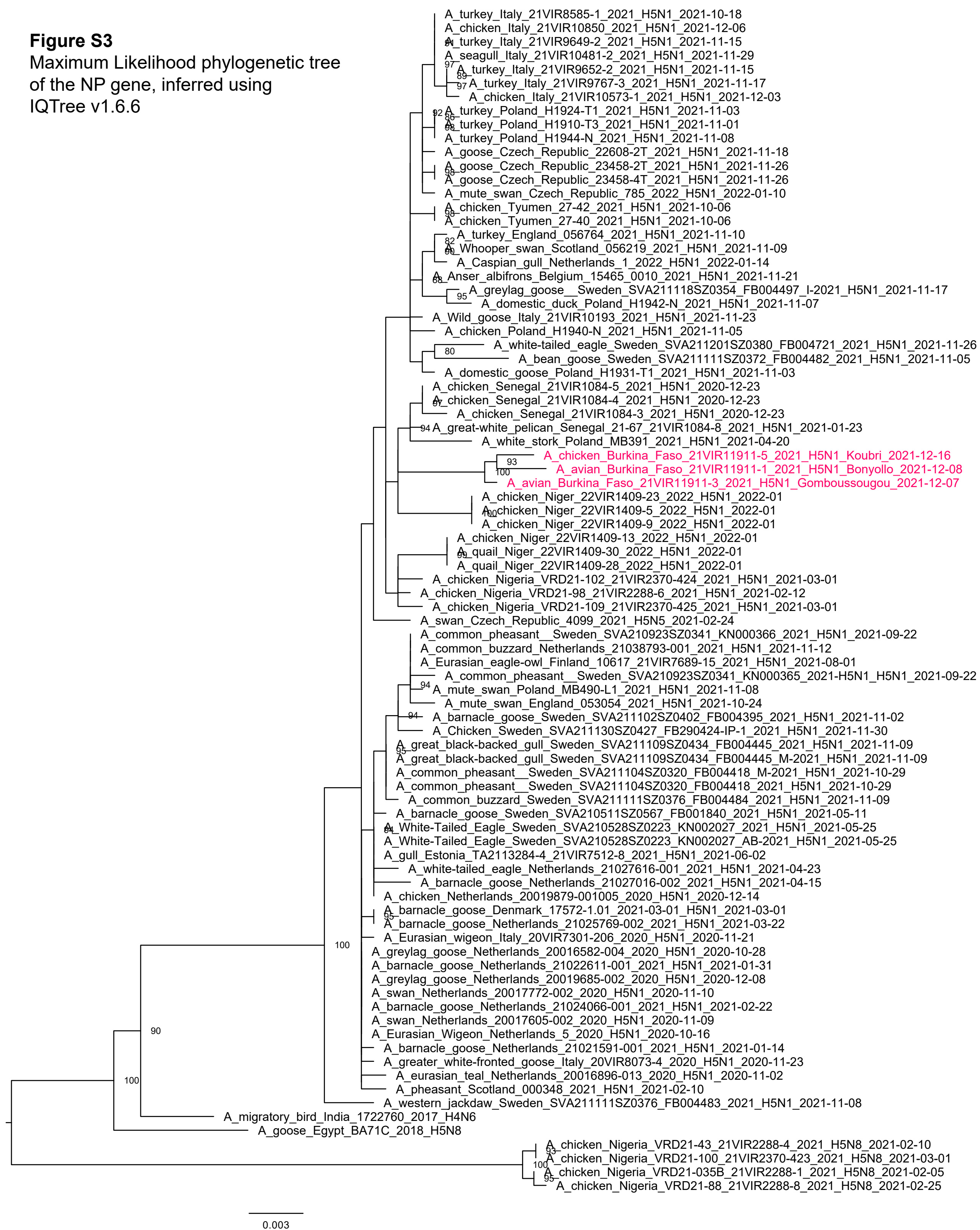

Supplement: Supplementary file 1 [file viruses-14-01901-s001.zip › Figure_S3_NP_tree.pdf]

**Figure S4**  
Maximum Likelihood phylogenetic tree  
of the NA gene, inferred using  
IQTree v1.6.6

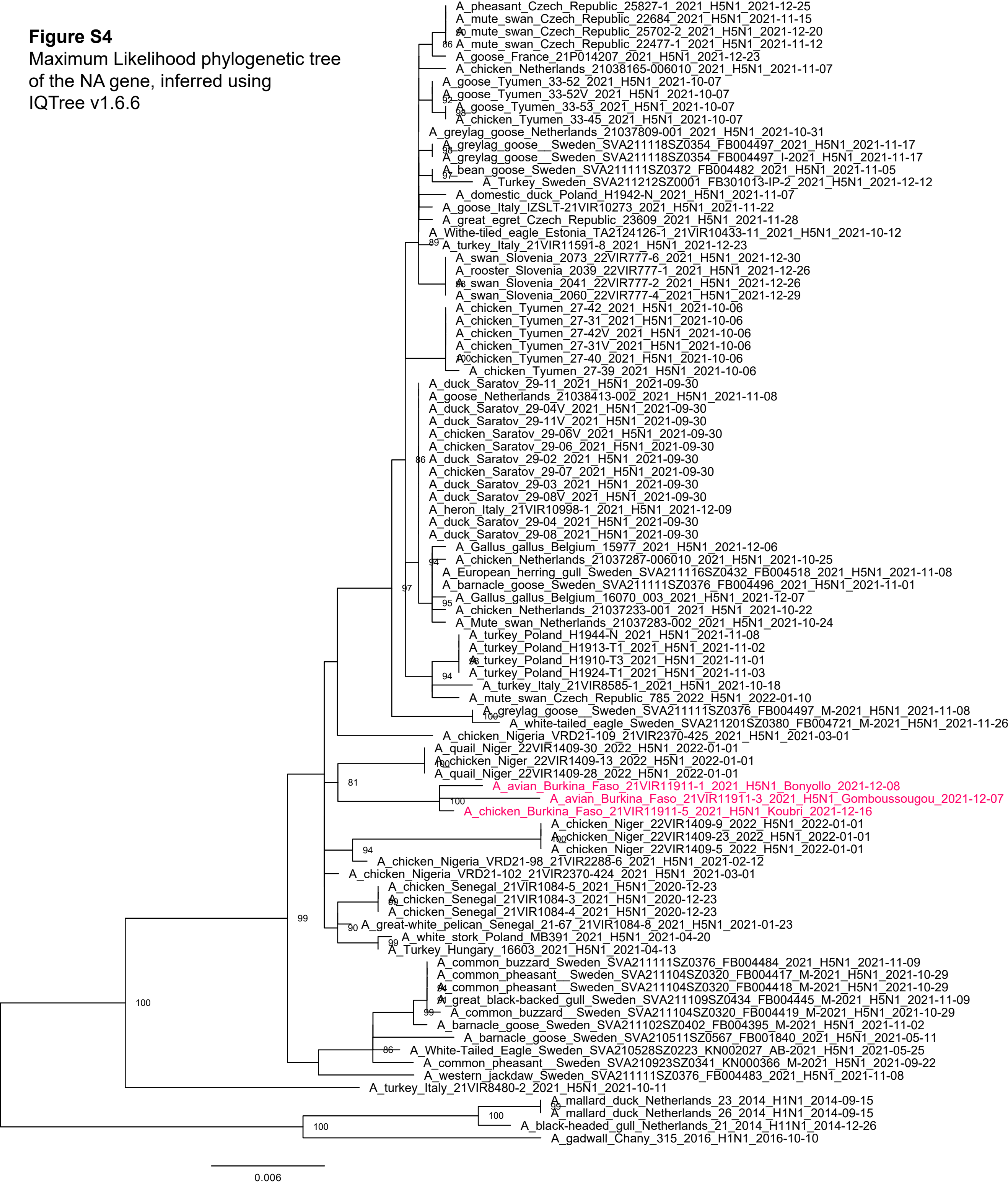

Supplement: Supplementary file 1 [file viruses-14-01901-s001.zip › Figure_S4_NA_tree.pdf]

Figure S5  
Maximum Likelihood  
phylogenetic tree of  
the M gene, inferred  
using IQTree v1.6.6

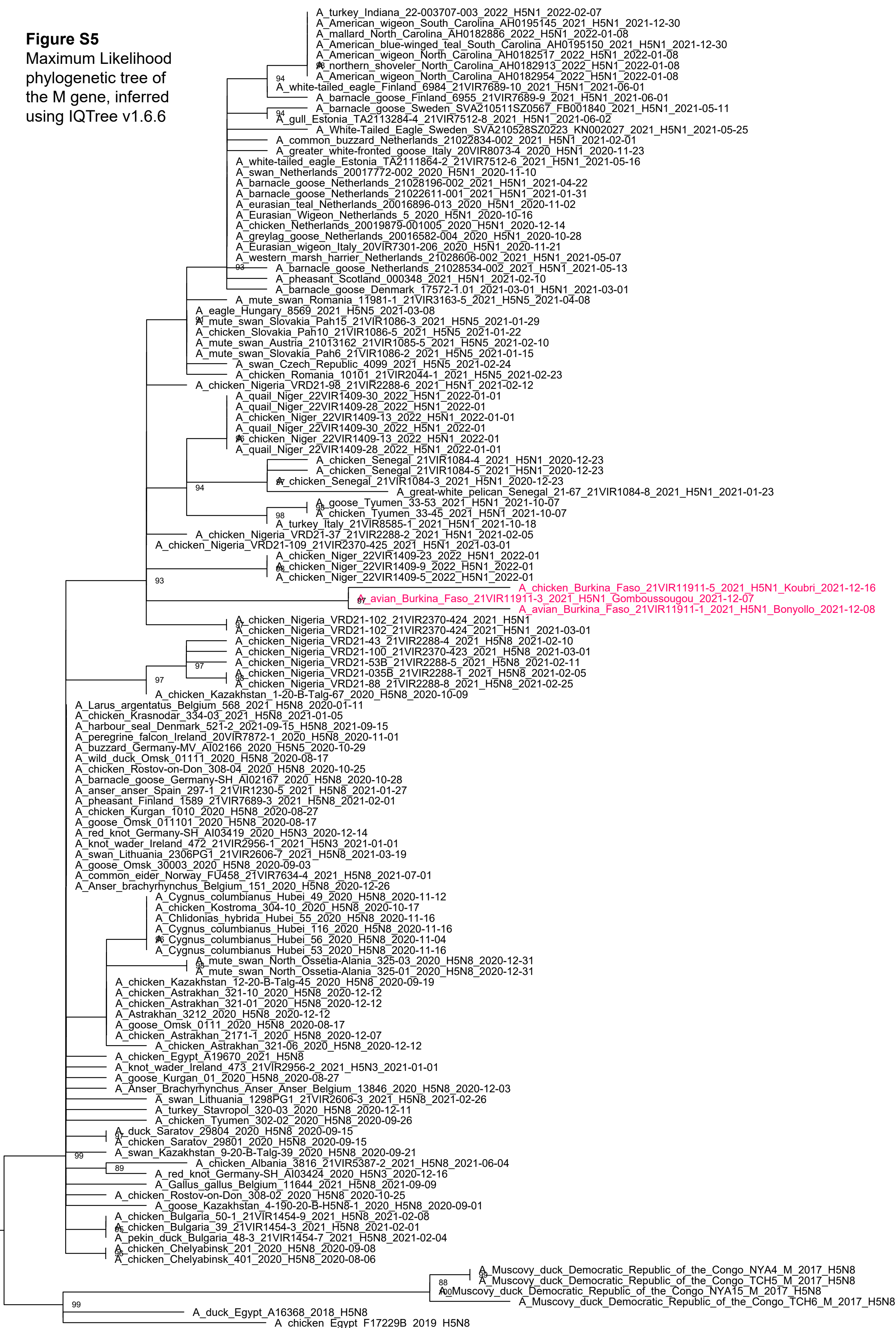

0.002

Supplement: Supplementary file 1 [file viruses-14-01901-s001.zip › Figure_S5_M_tree.pdf]

**Figure S6**  
Maximum Likelihood phylogenetic tree  
of the NS gene, inferred using  
IQTree v1.6.6

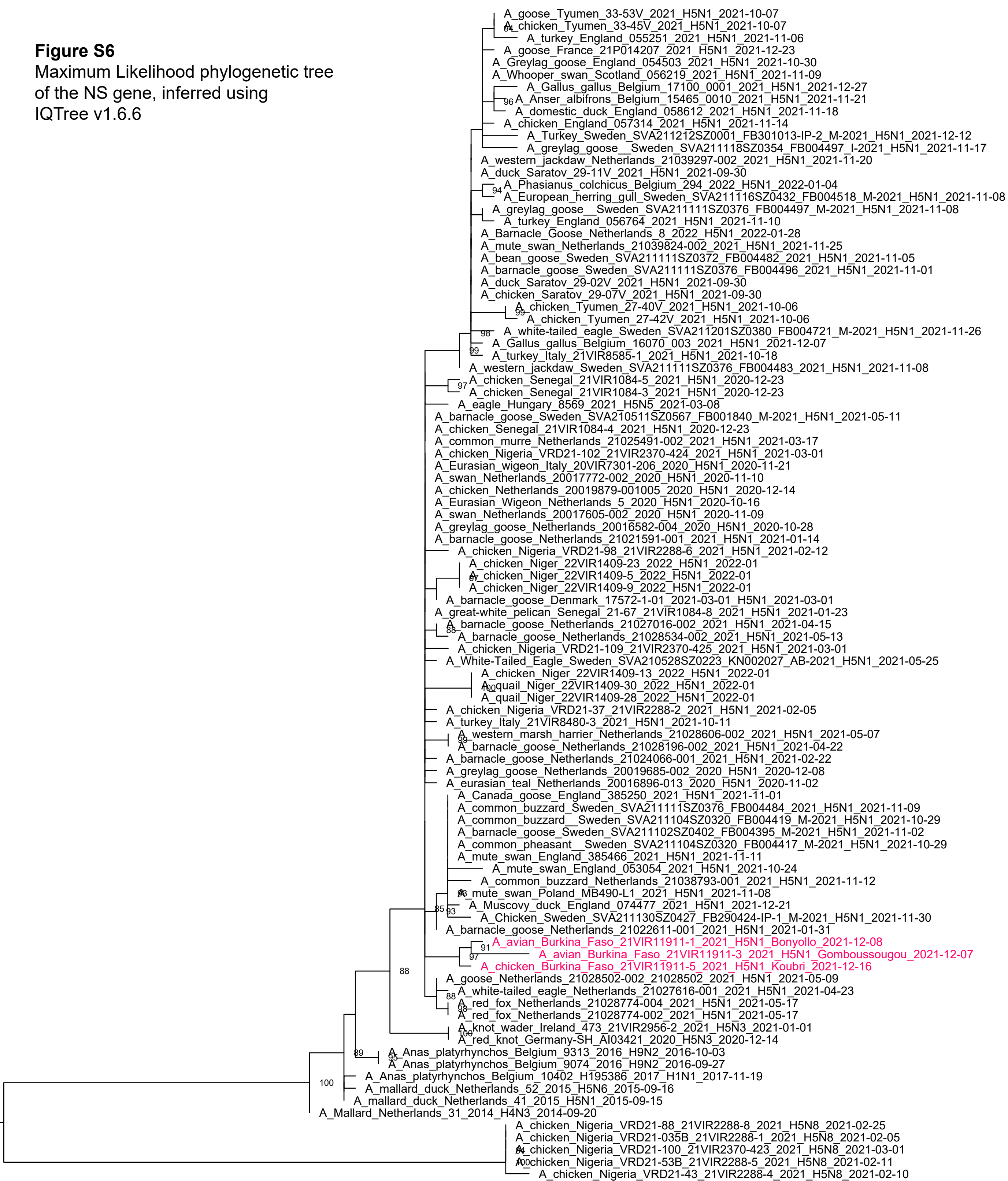

0.01

Supplement: Supplementary file 1 [file viruses-14-01901-s001.zip › Figure_S6_NS_tree.pdf]

**Figure S7**  
Bayesian Maximum Clade  
Credibility tree of the HA gene,  
inferred using BEAST v1.10.4

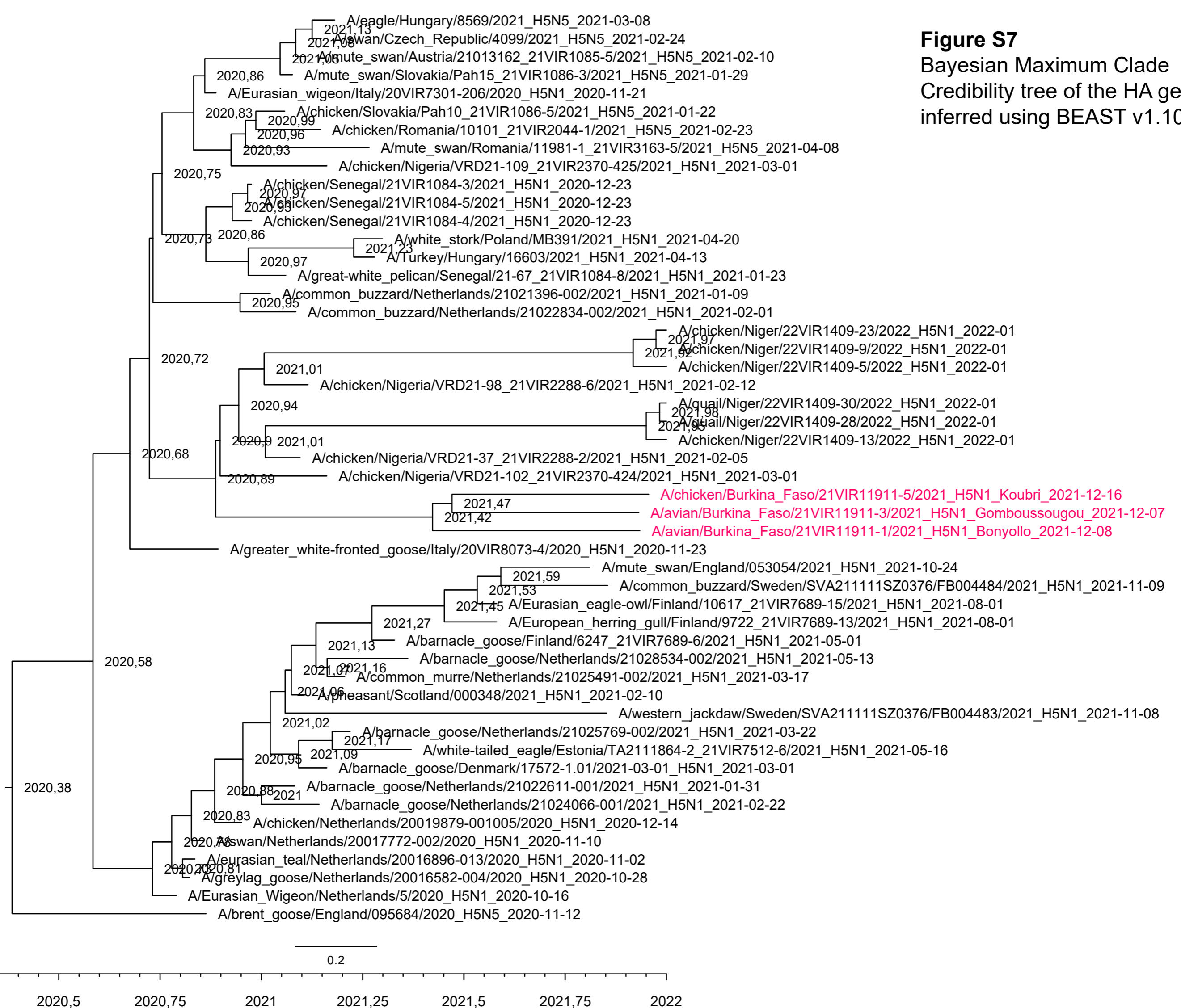

Supplement: Supplementary file 1 [file viruses-14-01901-s001.zip › Figure_S7_MCCtree.pdf]
